# Risk Mitigation of Pacemaker Pocket Erosion in Thin Patients

**DOI:** 10.1016/j.cjco.2022.03.002

**Published:** 2022-03-09

**Authors:** Bert Vandenberk, Kyle Murray, Jacques Rizkallah

**Affiliations:** a Department of Cardiac Sciences, Libin Cardiovascular Institute, Cumming School of Medicine, University of Calgary, Calgary, Alberta, Canada; b Department of Cardiovascular Sciences, KU Leuven, Leuven, Belgium

## Abstract

The aging population, particularly the thin and frail, has an increased risk of long-term cardiac implantable electronic device complications. This case is that of an elderly, thin-skinned patient who presented with a pacemaker pocket erosion 4 years after elective generator change, potentiated by a small pocket size with a superficial suture fixating the generator in the subcutaneous pocket. The risk for device erosion may have been mitigated during the generator change by increasing the size of the pocket, using a submuscular pocket, and potentially an absorbable antibacterial envelope. Fixation of the generator is considered optional.

## Résumé

Le risque de complications liées aux dispositifs électroniques cardiaques implantables est plus grand à long terme au sein de la population vieillissante, particulièrement chez les personnes minces et fragiles. Nous présentons ici le cas d’un patient âgé, à la peau fine, qui, quatre ans après le remplacement non urgent du générateur de son stimulateur cardiaque, a présenté une érosion de la loge du boîtier aggravée par la petite taille de celle-ci. Une suture superficielle fixait le générateur dans la loge sous-cutanée. Le risque d’érosion de la loge du boîtier aurait pu être atténué par l’augmentation de la taille de la loge lors du remplacement du générateur, par le recours à une loge sous-musculaire, et peut-être par l’utilisation d’une enveloppe antibactérienne résorbable. La fixation du générateur est jugée facultative.

The aging population will result in a global increase of patients aged 65 years or older from 6.9% up to 12.0% between 2000 and 2030.^1^ This change also will result in an associated increase in cardiac implantable electronic device (CIED) procedures. CIED procedures in the elderly, particularly in the thin and frail, may predispose these patients to an increased risk of procedural complications, including CIED infection and pocket erosion.^2^ We present a case of an elderly patient who presented with a pacemaker pocket erosion 4 years after elective generator change, potentiated by a small pocket size with a superficial suture fixating the generator in the subcutaneous pocket. Informed consent from the patient for publication of the case was obtained.

## Case

A 96-year-old female patient was referred to our centre for the management of a superficial, purulent pacemaker pocket erosion. Her cardiovascular medical history included persistent atrial fibrillation, arterial hypertension, and a body mass index of 15.4 kg/m^2^. In 2008, she received a single-chamber transvenous pacemaker for sinus node disease and paroxysmal atrial fibrillation. The initial implant procedure was aborted due to an acute left-sided hemopneumothorax requiring intensive-care-unit admission. The patient subsequently received a right-sided pacemaker. She underwent an elective generator change in 2017. Ever since the elective generator change in 2017, she had been aware of a superficial, hard nodule just caudal of the incision. Clinical examination revealed no signs of infection, and the nodule remained stable over time.

**Figure 1:**
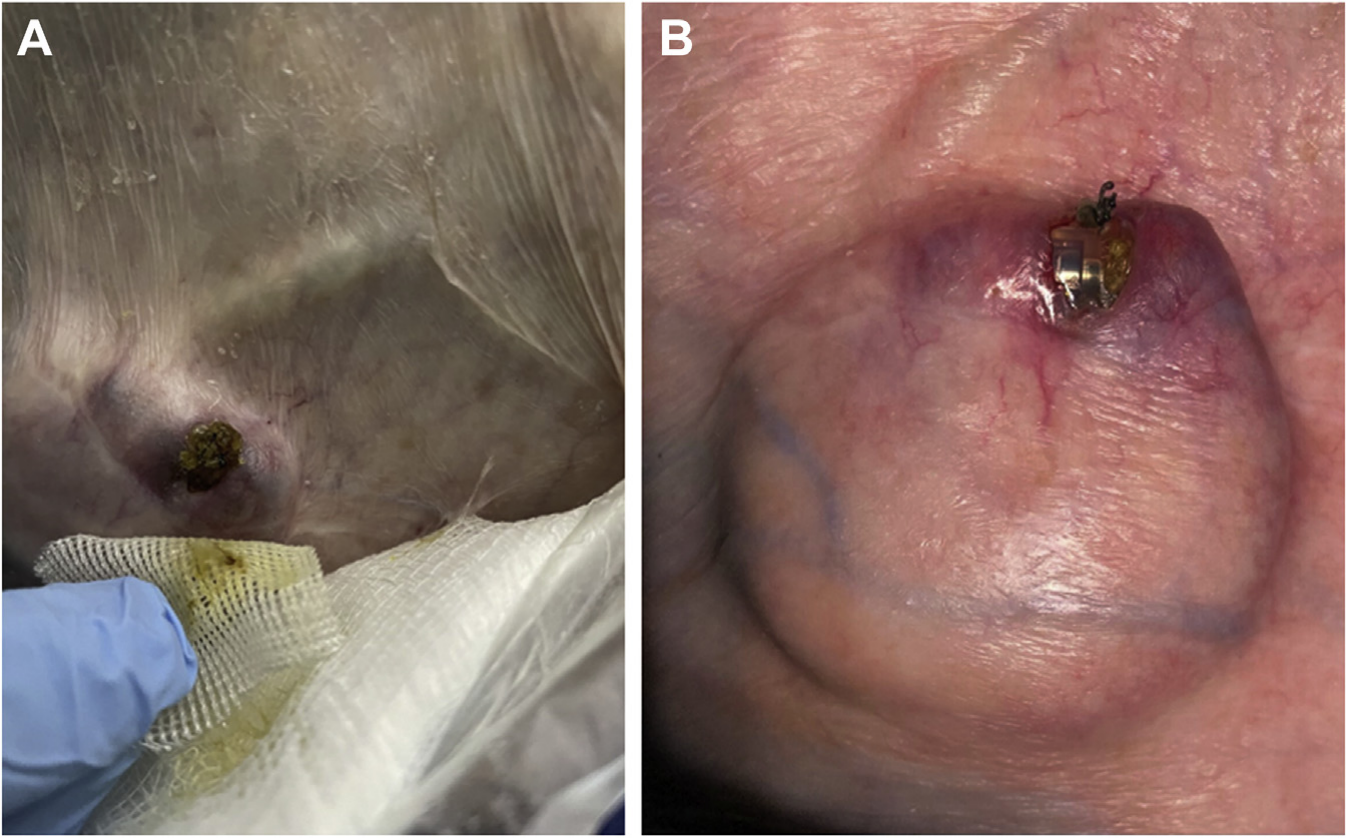
Pacemaker pocket erosion with skin deficit. (**A**) Site of device pocket erosion covered by granulation tissue (**B**) In the cleaned wound, the suture fixating the generator in the subcutaneous pocket is visible as a potential contributor to the pocket erosion. The patient’s thin skin and sparse subcutaneous tissue can be appreciated given the visible outline of the pacing lead superior to the generator.

About 6 weeks before the referral, she noticed a superficial wound erosion with purulent excretion over her pacemaker pocket. Her family physician prescribed a 2-week antibiotic treatment with trimethoprim/sulfamethoxazole. Despite the antibiotics, the erosion progressed (Fig. 1A), and after cleaning of the wound, a skin deficit was noted originating around a polypropylene suture with the pacemaker generator visible underneath the skin (Fig. 1B). The diagnosis of a CIED generator erosion with pocket infection was made. Lead measurements upon device interrogation were within normal range. The ventricular pacing percentage was 62.7% while programmed with ventricular demand pacing at a lower rate of 60 beats per minute.

The patient denied infection-related symptoms, such as fever, chills, and night sweats. Biochemistry showed a normal white blood cell count (4800/μL), C-reactive protein level (3.4 mg/L), and high-sensitive C-reactive protein level (3.0 mg/L). Peripheral blood cultures remained negative. However, cultures of the pocket from within the erosion site were positive for methicillin-sensitive *Staphylococcus aureus*. A transthoracic echocardiogram with good visualization showed a normal left and right ventricular function with pulmonary hypertension of 75 mm Hg. The right ventricular lead was carefully assessed, and no vegetations were identified.

Further diagnostic and treatment options were discussed with the patient, her family, cardiac anesthesiologists, and the infectious disease department. Given the low likelihood of lead endocarditis and the overall frailty of the patient, a decision was made to explant the device with cutting and intravascular retraction of the right ventricular lead through a separate incision, rather than performing a complex lead extraction. The procedure was uneventful, and after deep cultures were obtained, the patient was started on antibiotic treatment with cefazolin. She was not pacemaker-dependent, but she did have intermittent symptomatic bradycardia. Therefore, no temporary pacemaker was required to bridge the time to reimplant. Given the absence of systemic infection, a leadless pacemaker was implanted 1 week later. The patient was transferred back to the referring hospital to complete her 6-week antibiotic treatment, which included 2 g of cefazolin given intravenously every 8 hours, and 100 mg doxycycline twice a day. Afterward, lifelong antibiotic suppression adjusted for renal function was initiated with 500 mg cephalexin once daily.

## Discussion

In the case presented, the etiology of the CIED erosion late after elective generator change was believed to be due to multiple factors, including the patient’s underweight with thin tissue layers, advanced age, the superficial suture fixating the generator in the subcutaneous pocket, and poor accommodation of the new pacemaker generator in the small device pocket.

Which of these factors was the main contributor to the eventual device erosion is not clear, but the central position of the suture fixating the generator in the device erosion suggests a causal relationship. According to the 2021 European Heart Rhythm Association expert consensus statement on CIED implant techniques, generator fixation is optional, and no recommendations on suture material or technique are provided.^3^ Generator fixation can be considered in cases of a wandering generator, or when a history of Twiddler syndrome is present. On the other hand, generator fixation has been shown to be associated with increased risk of pocket hematomas in elderly patients on oral anticoagulation therapy.^4^ Alternatively, a submuscular pocket should be considered in patients at risk for device erosion, particularly in underweight patients with a pectoral muscle that is itself thin and easily accessible.^3^ Lastly, this patient might have benefited from the use of an absorbable antibacterial envelope in the prevention of pocket infection.^5^ Very limited data suggest that earlier nonabsorbable pouches reduced the risk of Twiddler syndrome recurrence in patients with CIED revision for Twiddler syndrome, but to our knowledge, no evidence shows that absorbable pouches have a similar effect.^6^

Further, aging itself is a major risk factor for CIED-related complications, often due to coinciding comorbidities. Aging is associated with a loss of subcutaneous fat and body fat redistribution; both may result in the thin-skinned appearance seen in the elderly.^7^^,^^8^ The lack of sufficient subcutaneous fat may result in a constant outward pressure projected by the CIED components, including the generator, the leads, or both, against the skin, which is the last barrier of protection around the implanted device. This pressure may lead to progressive thinning of the skin with device protrusion, and eventually erosion with externalization of the CIED components. Therefore, the risk of device erosion in thin-skinned patients can be minimized by creating a sufficiently large device pocket, which reduces the pressure on the skin, considering a submuscular pocket for the generator, or implanting a leadless pacemaker instead of a transvenous system (if applicable).

The management of the case deviates from standard recommendations.^5^ First, investigations to rule out lead endocarditis were limited. No transesophageal echocardiogram or positron emission tomography/leukocyte scan was performed; therefore, lead endocarditis was not formally excluded prior to intervention. Second, the intervention of choice would be a complex lead extraction with complete CIED system removal.^5^ However, given the increased procedural risk related to the right-sided device, the 13-year-old lead, and patient frailty, a joint decision was made to cut the lead under the right clavicle with intravascular retraction. This option is in fact the least favourable, as this makes a future percutaneous extraction attempt more difficult, requiring lead snaring. Further, this typically mandates lifelong antibiotic suppression. Therefore, we believe that the treatment options should be discussed with an experienced extraction centre prior to deciding upon cutting a lead with intravascular retraction.

## Conclusion

Frail patients are at increased risk of long-term CIED complications, such as pocket erosion. Upon elective generator replacements, this risk should be mitigated by a careful assessment of the pocket. Adjustment of the pocket size and use of a submuscular pocket should be considered, particularly in underweight patients.

**Novel Teaching Points**

• Pacemaker pocket erosion is uncommon, but elderly, frail patients are at increased risk due to loss of subcutaneous fat and fat redistribution.
• The risk for device erosion could be mitigated during a generator replacement by increasing the size of the pocket, using a submuscular pocket, and potentially, using an absorbable antibacterial envelope.
• Fixation of device generators is considered optional.
